# Personal ultraviolet Radiation exposure in a cohort of Chinese mother and child pairs: the Chinese families and children study

**DOI:** 10.1186/s12889-019-6610-y

**Published:** 2019-03-08

**Authors:** Michael G. Kimlin, Liwen Fang, Yajing Feng, Linhong Wang, Ling Hao, Jing Fan, Ning Wang, Fanwen Meng, Ruilan Yang, Shu Cong, Xiaofeng Liang, Baohua Wang, Martha Linet, Nancy Potischman, Cari Kitahara, Ann Chao, Yu Wang, Jiandong Sun, Alison Brodie

**Affiliations:** 1 University of the Sunshine Coast, Maroochydore, Faculty of Science, Health, Education and Engineering, Locked Bag 4, Maroochydore DC, Queensland 4558 Australia; 2 Cancer Council Queensland, Brisbane, Australia; 3 National Center for Chronic and Noncommunicable Disease Control and Prevention, Chinese Center for Disease Control and Prevention, 27 Nanwei Road, Xicheng District, Beijing, 100050 Beijing China; 4 US Centers for Disease Control and Prevention, Atlanta, GA USA; 5 Laoting County Maternal and Child Health Hospital, Laoting, Hebei China; 6 Taicang County Maternal and Child Health Hospital, Taicang, Jiangsu China; 7 Chinese Center for Disease Control and Prevention, Beijing, China; 8 National Cancer Institute (NIH) - Division of Cancer Epidemiology and Genetics, Bethesda, MD USA; 9 National Cancer Institute, Office of Dietary Supplements, Bethesda, MD USA; 10 National Cancer Institute, Center for Global Health, Bethesda, MD USA

**Keywords:** Chinese, Cohort, Dosimetry, Mothers and child pairs, Ultraviolet radiation

## Abstract

**Background:**

Few studies in China have examined personal ultraviolet radiation (UVR) exposure using polysulfone dosimetry.

**Methods:**

In this study, 93 mother and adolescent child pairs (*N* = 186) from two locations in China, one rural (higher latitude) and one urban (lower latitude), completed 3 days of personal UVR dosimetry and a sun/clothing diary, as part of a larger pilot study.

**Results:**

The average daily ambient UVR in each location as measured by dosimetry was 20.24 Minimal Erythemal Doses (MED) in the rural location and 20.53 MED in the urban location. Rural mothers had more average daily time outdoors than urban mothers (5.5 h, compared with 1.5 h, in urban mothers) and a much higher daily average personal UVR exposure (4.50 MED, compared with 0.78 MED in urban mothers). Amongst adolescents, rural males had the highest average daily personal UVR exposure, followed by rural females, urban females and urban males (average 2.16, 1.05, 0.81, and 0.48 MED, respectively).

**Conclusions:**

Although based on small numbers, our findings show the importance of geographic location, age, work/school responsibilities, and sex of the adolescents in determining personal UVR exposure in China. These results suggest that latitude of residence may not be a good proxy for personal UVR exposure in all circumstances.

## Background

Ultraviolet radiation (UVR) has a significant impact on human health. Underexposure to UVR results in limited opportunity for vitamin D synthesis and can lead to vitamin D deficiency and associated conditions (bone weakness, skeletal deformities, muscle aches, and cardiovascular disease and possibly some cancers) while overexposure to UVR may suppress cell-mediated immunity and increase the risk of developing skin cancers and eye diseases such as cataracts [1]. There are many factors that influence the amount of UVR exposure that an individual receives, including environmental factors (geographic location of residence, season, humidity, ozone, pollution), host factors (skin color, eye color, etc), demographic factors (age, sex, race and others), occupational factors (employment status and occupation type), and potentially modifiable behavioral factors (time spent outdoors, clothing worn while outdoors) [2–6].

Perhaps because the incidence of skin cancer in China is much lower than in countries with fair-skinned Caucasian populations [7], there are a limited number of Chinese studies examining UVR exposure in relation to skin cancer risk. By contrast, the high prevalence of hypovitaminosis D across all ages in China [8, 9] has prompted several studies investigating UVR exposure as a determinant of vitamin D production, and vitamin D knowledge, attitudes and behavior. In these studies, UVR exposure is commonly assessed by questionnaire [10–13] or is estimated using local ambient UVR (latitude) as a proxy for personal UV exposure [14–16]. There have been few studies in China that have used objective measures of UVR exposure, such as personal UVR dosimetry [17, 18].

Polysulfone dosimetry is a convenient, cost-effective, objective means of measuring UVR exposure. When temporally combined with a sun exposure diary describing clothing worn and time outdoors, it is the gold standard for measurement of an individual’s personal UVR exposure. This paper will overview the methods, findings, functionality and limitations of using personal UVR dosimetry and a sun diary questionnaire in a pilot study of Chinese mothers and their teenage children conducted in two geographic regions in China. It will 1) provide information on how latitude, occupation, age, sex and urban/rural residence impact on UVR exposure and sun protective behavior, and 2) assess the feasibility of this type of data collection and consider what modifications may be required before roll-out of the study on a large scale.

## Methods

### Overview

The Chinese Children and Families Cohort Study (CFCS) was envisaged as a follow-up of the 240,000 offspring and their families who participated in the 1993–1995 Community Intervention Program (CIP) of folic acid supplementation for the prevention of neural tube defects. Prior to launching a wider-scale follow-up of the entire study population to assess the late effects of periconceptual folic acid, two pilot studies were conducted: the first to assess the ability to reconstruct and follow-up the CIP cohort by tracing and interviewing a sample of the original study participants (mother, father, and child) [19], and the second to examine the feasibility of collecting extensive diet, physical activity and UVR exposure data in a subset of the first pilot study population [20]. The second pilot study included 93 mothers and 93 adolescent children aged 14–17 years, with approximately half from one urban location in south-eastern China (latitude 31.3 N) and half from one rural location in north-eastern China (latitude 39.3N), both geographic regions where the 1993–1996 CIP was carried out. Between April and June 2012, over an eight day period during the school term, participants completed a food frequency questionnaire and food diary, a physical activity questionnaire, a three-day sun exposure diary together with 3 days of personal UVR polysulfone dosimetry measurements, seven days of pedometry measurements, and provided blood, saliva, and toenail samples. Grip strength and body composition measurements were also taken and ambient solar UVR was monitored in both study locations. This paper describes the methods and findings of the solar UVR measurement component of the CFCS pilot study.

### Ethics, consent and permissions

The Institutional Review Boards (IRBs) and ethics committees of the participating institutions, the Chinese Center for Disease Control and Prevention (China CDC; protocol 201,110), United States (US) Centers for Disease Control and Prevention (CDC, protocol 6140), and the US National Cancer Institute (NCI, protocol 11CN165), approved the project before data collection began and have since obtained renewed IRB approval annually. Prior to data collection, all mothers, and children aged 18 years and over, signed written consent to participate in the study. Children aged under 18 years provided signed Assent, and their parents provided written consent for them to participate in the study.

### Fieldwork preparation

A research team (Kimlin, Brodie and Sun) with expertise in measurement of solar UVR developed the fieldwork protocol and conducted three-day training and practice workshops in the rural and urban study locations. The UVR experts trained the local Maternal and Child Health Hospitals (MCH) staff interviewers in the measurement procedure. Verbal and written instructions to MCH interviewers were provided in Chinese. Approximately one month after the training, supervisory staff from China CDC returned to each study site to oversee the data collection, and monitor and re-train the staff as needed on various aspects of the data collection protocol [20].

### UVR dosimetry

Polysulfone dosimetry is the gold standard for measuring solar UVR exposure in the wavelength range from 295 to 320 nm (UVB) [21], and polysulfone dosimeters were used in this study to assess participants’ personal UVR exposure. The dosimeters were prepared and provided by the UVR experts from the Queensland University of Technology (QUT) in Brisbane, Australia. To create the dosimeters, an aqueous polysulfone gel solution was carefully poured onto a smooth glass casting table, where a casting blade created a polysulfone film approximately 40 μm thick. The resulting film was allowed to set, carefully cut and affixed to a rigid plastic holder with a central circular aperture where the clear film is exposed. Each piece of polysulfone film may have small variations in thickness and smoothness, and these small differences may affect how the film will absorb UVR. For this reason, every dosimeter was individually measured before use in fieldwork, by recording the optical absorbance of the dosimeter at 330 nm, using a UV spectrophotometer.

Dosimeters were supplied to the field precoded, premeasured, and standardized with a calibration traceable to the world UV standard (variability between batches was less than 10%). Three dosimeters were provided per participant – one for each of the three days of UVR measurement. Dosimeters were replaced daily to avoid potential saturation with UVR. Individual dosimeters were stored in opaque envelopes to prevent incidental exposure to UVR. Dosimeters were worn daily for each of three consecutive days (Sunday to Tuesday), while participants went about their normal activities of daily living. Upon waking, each participant was instructed to carefully remove a dosimeter from its individual packet (taking care to avoid touching the clear polysulfone film) and attach it to an expandable band (provided) by a sticky Velcro® dot. Participants were instructed to keep the dosimeter band freely exposed to the sun at all times during the day. At the end of the day, before retiring, the dosimeter was to be removed and replaced back into the opaque packet.

**Fig. 1:**
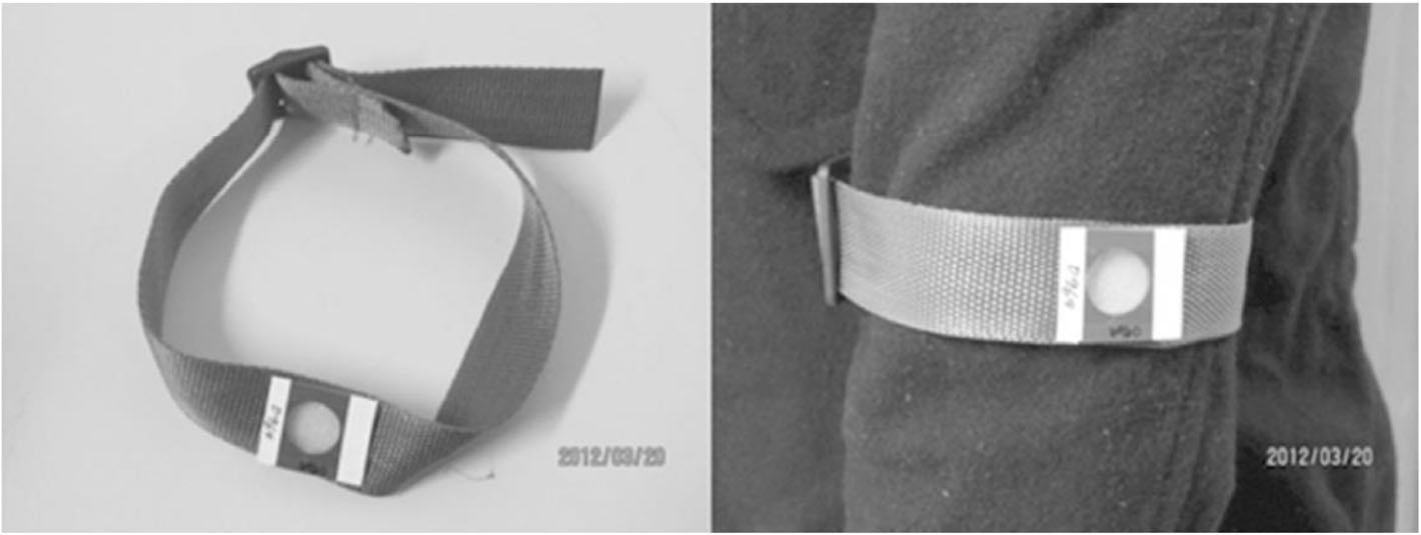
Polysulfone dosimeter mounted on adjustable band, Chinese Families and Children Study, 2012

Trainers initially instructed that the dosimeter bands be worn on the upper arm, however local doctors who field-tested this position during training suggested that the wrist would be a more convenient location, as the dosimeter would be less likely to be forgotten when adding or removing layers of clothing. Given the importance of ensuring the dosimeter was worn throughout the day, training staff decided prior to the commencement of data collection that the dosimeter bands should be worn at the wrist level, over the outer layer of clothing. Various types of band were tested as dosimeter bands, including a hospital paper wristband, and a wide elastic band. The band finally chosen was adjustable, non-elastic, and designed by the field staff to allow a firm fit against skin or clothing (see Fig. 1). At the beginning and end of each dosimeter use, participants recorded the ‘Time on’ and Time off’ on each dosimeter packet. Any problems encountered in wearing the dosimeter (i.e. it became wet or was taken off along with outerwear and not replaced) were recorded on the sun diary described below. All dosimeters were collected after the three days of wear.

### Sun exposure diary

A three-day sun exposure diary was completed in conjunction with the three days of personal UVR dosimetry. The sun diary instructed respondents to record, on an hourly basis from 5 am to 7 pm, whether and for how long they were outdoors, and their clothing worn. In addition, each participant was asked to record use of sun-protective measures such as hats, masks, scarves, sunglasses, sunscreen and umbrellas. For each hourly interval, participants: ticked a box corresponding to the amount of time in minutes they spent in the sun (0, < 15, 15–29, 30–44, 45–60 min); circled a number corresponding to the headwear, clothing worn on the upper and lower body, and footwear they wore (with reference to a supplied clothing guide showing photographs); ticked a box to indicate whether they wore sunscreen or products containing sunscreen, gloves, sunglasses or an umbrella, and shaded a diagram to indicate where on the body that sunscreen (if any) was worn. For this study, a validated sun diary was adapted from a version used with good compliance in previous large UVR exposure studies [22, 23], and was translated into Chinese. Photos from the clothing guide attached to the diary were replaced to account for cultural differences in clothing between Australia and China. Local study interviewers assisted participants with diary completion on Day 1, thereafter participants completed the diaries themselves. The combination of diary and dosimeter provides a detailed record of an individual’s personal UVR exposure.

### Ambient UVR

It was important to measure the available ambient (environmental) UVR in each location during the study period in order to provide information on the maximum available UVR exposure for the days of the study. UVR dosimetry has an advantage over satellite data for measuring ambient UVR as it is more reflective of the local environment, taking into account clouds, aerosols and local variations. For ambient UVR measurement, a single polysulfone dosimeter (the same as worn by the study participants) was placed in an unobstructed position on the roof of a central MCH hospital at each study location, where it was freely exposed to full sunlight throughout the day. Dosimeters were placed and changed each day after sunset in preparation for measuring ambient UVR starting at sunrise the following day. Ambient UVR was measured twice in each location, for seven consecutive days during the first and last weeks of each data collection period. Measures were made in the urban region in the southeast from April 9–15 and May 28–June 3, 2012 and in the rural region in the northeast from 20-27 May and 10–16 June, 2012.

All dosimetry and diary data were reviewed by the UVR measurement supervisory team, which included Chinese and US study investigators involved with training, data collection and data entry/analysis, and the Australian UVR research team who provided the dosimeters and training, at a data management workshop at the China CDC in Beijing in 2013. Decisions were made to standardize data coding, data linkage, and analysis. Diary data was double-entered and anomalies checked against the original hard copies, and corrected.

### Laboratory, questionnaire, and statistical analysis

#### Dosimeters

All used polysulfone dosimeters were collected and returned to a laboratory in Brisbane, Australia, for post-measurement analysis. Here, dosimeters were carefully cleaned with ethanol to remove dirt or dust, then were measured for their post-intervention spectrophotometric absorbance at 330 nm (A330) using the same procedure as for pre-measurement. The pre-to-post change in A330 of each dosimeter was calculated and a calibration factor was applied to ensure that the dosimeter readings were stable. Daily personal UVR exposure from the dosimetry data was averaged over the number of days that dosimeters were worn. For over 90% of participants, this was a three-day average. The resultant value represents the cumulative daily UVR exposure, expressed as Minimal Erythemal Dose (MED) or Joules per meter squared; 1 MED = 210 J/m2.

Average ambient UVR was calculated for each location by averaging the 14 daily measures obtained from the dosimeters exposed on the hospital roofs – 7 measures from the first week of data collection in that location, and 7 from the last week.

#### Sun diary

Total daily time outdoors was calculated from the checked boxes on the sun diary, by summing the midpoint values (0, 7.5, 22.5, 37.5, 52.5 min, respectively) of each of the five time categories (0, < 15, 15–29, 30–44, 45–60 min) at each of the 14 hourly intervals each day (5 am to 7 pm), using an established protocol [4]. The daily values for each participant were then summed and averaged over the number of days in which diaries were completed.

## Results

In both locations, there was high (over 90%) compliance with wearing the dosimeters and completing the sun diary (Table 1). Five urban mothers and two urban adolescents did not complete any dosimetry, while 5 rural participants (3 mothers and 2 adolescents) completed less than the full three days of dosimetry. In some cases, this was because dosimeters became detached from their bands and were lost. Eight urban participants and four rural participants completed less than three days of the sun diary.

**Table 1:** Compliance with UV dosimetry and sun diaries, Chinese Families and Children Study, 2012

	Urban			Rural		
	Mother *n* = 52	Child *n* = 52	Total *n* = 104	Mother *n* = 41	Child *n* = 41	Total *n* = 82
UVR DOSIMETRY						
Completed 3 days	47	50	97	38	39	77
Completed 2 days	–	–	–	3	1	4
Completed 1 days	–	–	–	–	1	1
Completed 0 days	5	2	7	–	–	–
SUN DIARY						
Completed 3 days	48	48	96	38	40	78
Completed 2 days	1	3	4	3		3
Completed 1 day	2	1	3	–	1	1
Completed 0 days	1	–	1	–	–	–

Individual level data were available for 186 participants, including 93 mothers and 93 adolescents (47 boys and 46 girls). Among all participants, 82 (41 mothers and 41 adolescents) were from the rural north-eastern location and 104 (52 mothers and 52 adolescents) were from the urban south-eastern location.

Participant characteristics have been described previously [20]. Briefly, mothers in the urban location were on average three years younger (38.7 years) than the rural mothers (41.5 years) (Table 2).

**Table 2:** Characteristics of the study participants, Chinese Families and Children Study, 2012 [20]

	Urban (south-eastern location)			Rural (north-eastern location)		
	Mothers *n* = 52	Adolescents *n* = 52		Mothers *n* = 41	Adolescents *n* = 41	
	F	M *n* = 33	F *n* = 19	F	M *n* = 14	F *n* = 27
Number of participants (%)	52	33 (63%)	19^a^ (37%)	41	14 (34%)	27 (66%)
Average age in years (range)	38.7 (35–44)	15.1 (14–17)	15.1 (14–16)	41.5 (36–54)	15.1 (14–17)	15.4 (14–16)
Average height in cm (range)	157 (142–169)	171 (159–184)	162 (156–172)	157 (145–166)	170 (165–174)	161 (146–172)
Average weight in kg (range)	55 (41–76)	64 (49–92)	55 (44–84)	63 (42–86)	62 (48–83)	58 (44–78)
Average BMI kg/m^2^ (range)	22.4 (16.2–30.6)	21.9 (17.2–34.0)	21.0 (16.9–30.2)	25.5 (18.9–37.3)	21.5 (16.0–27.8)	22.3 (17.2–29.4)
Currently attending school^b^ (%)	3 (6)	29 (88)	17 (89)	1 (2)	12 (86)	27 (100)
Working (%)	43 (83)	2 (6)	1 (5)	9 (22)	1 (7)	–
Neither attending school nor working (%)	4^c^ (8)	2 (6)	1 (5)	31 (76)	1 (7)	–
Never smoked (%)	52 (100)	33 (100)	18 (95)	41 (100)	14 (100)	27 (100)
Current alcohol consumption (%)	48 (92)	18 (55)	4 (21)	40 (98)	6 (43)	1 (4)

^a^ Missing data on one girl – *n* = 18 for these variables

^b^ With or without work

^c^Missing information for two mothers

Nine of 41 (22%) of the rural mothers worked (mostly in field and greenhouse farming), 1/41 (2.4%) attended school, and 75.6% neither worked nor attended school. By comparison, 43/52 (86%) of urban mothers worked, 3/52 (5.8%) attended school, and 4/52 (7.7%) neither worked nor attended school. In both locations, almost all male and female offspring reported currently attending school.

The study locations were separated by 8 degrees of latitude (Table 3). The rural location has a monsoon-influenced humid continental climate, with four distinct seasons, while the urban location has a humid subtropical climate with significant rainfall throughout the year. At the time of the year in which data was collected (April to June), the mean monthly temperature and humidity ranges from 11.5 to 22 C (56–73% humidity) in the north-eastern rural location and from 14.8 to 23.8 C (75–82% humidity) in the south-eastern urban location (China Meteorological Association).

**Table 3:** Environmental characteristics of the study locations, Chinese Families and Children Study, 2012

	Urban	Rural
Latitude	31.3° N	39.3° N
Average ambient solar UVR (MED^a^, April–June)	20.53	20.24
Average humidity^b^		
April	75%	58%
May	74%	63%
June	82%	73%
Average (daily mean) temperature (April–June)		
April	14.8°C	11.5°C
May	20.4°C	17.3°C
June	23.8°C	22.0°C

^a^Minimal Erythemal Dose

^b^Average data for the period 1971–2000, China Meteorological Data Service Center, http://data.cma.cn/en [40]

**Fig. 2:**
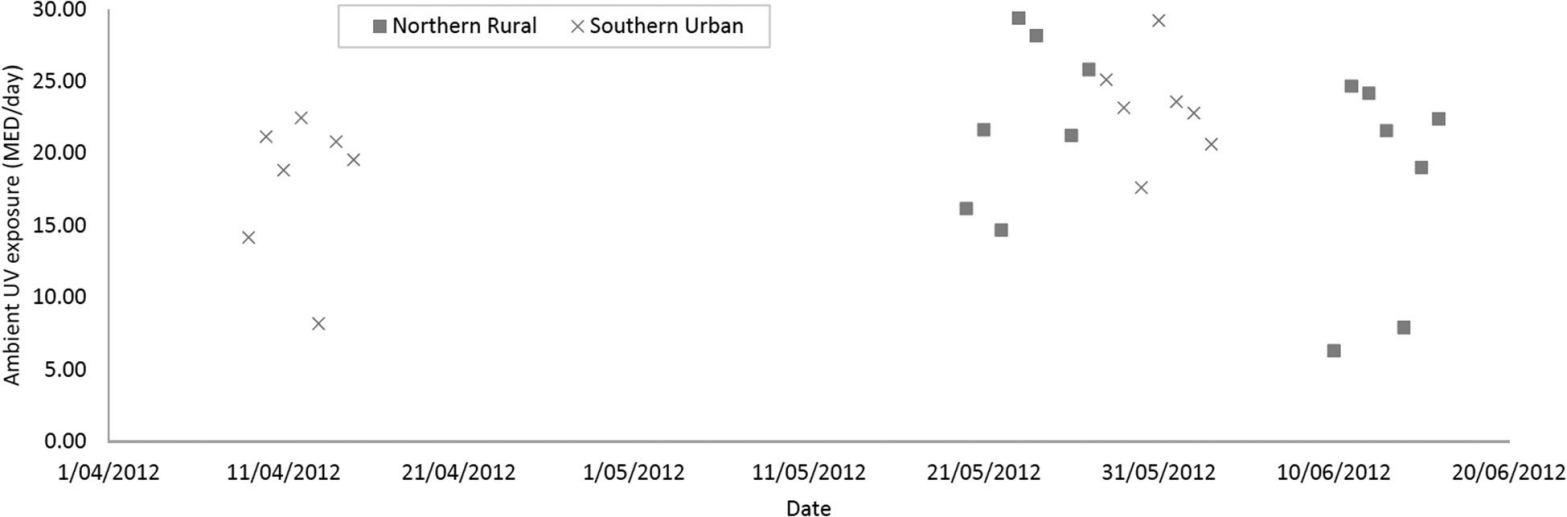
Daily ambient ultraviolet radiation (MED) in northern rural and southern urban locations, Chinese Families and Children Study, 2012

**Fig. 3:**
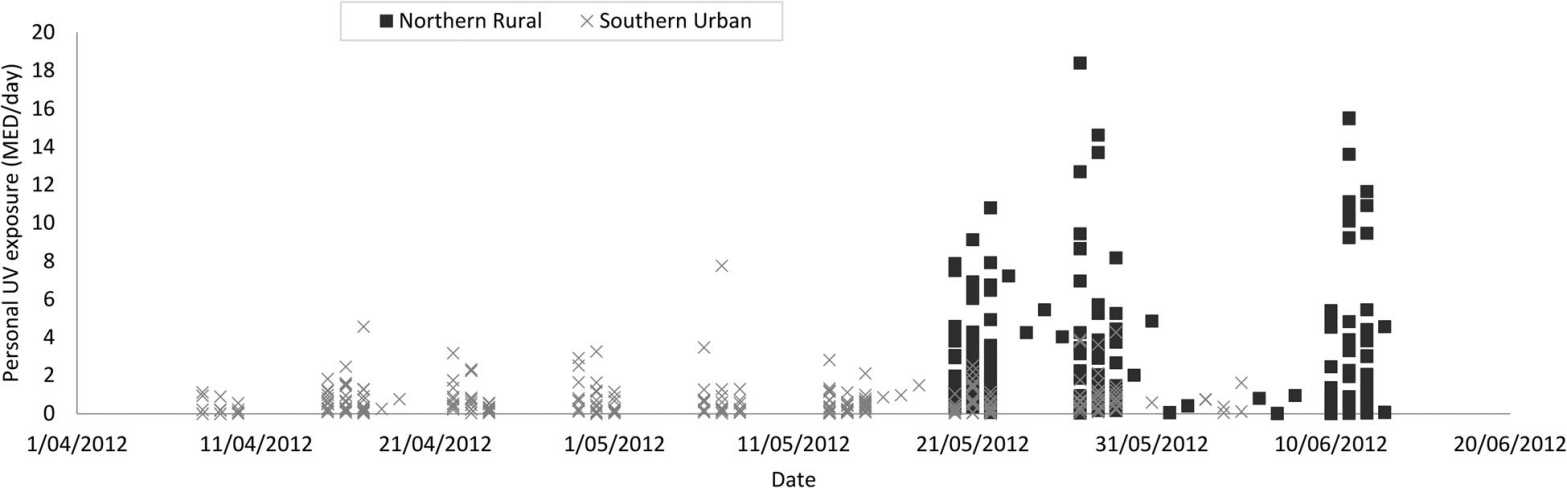
Daily personal ultraviolet radiation exposure dose (MED) of participants in northern rural and southern urban locations, Chinese Families and Children Study, 2012

Daily ambient UVR, measured by dosimetry, ranged from 8.2 to 29.22 in the urban location, and from 6.31 to 29.4 in the rural location (Fig. 2). The average ambient solar UVR over the period of data collection was almost identical in both locations (rural 20.24 MED, urban 20.53 MED). Because ambient UVR increases from April to June and the measures were not made at the same time in both locations, the averages were not completely comparable. However, the change in ambient UVR appeared to be gradual over the data collection period (Fig. 2) and therefore the average ambient UVR values in each location would be expected to be very similar, if daily measures were made over the entire period of data collection. Ambient UVR and personal UVR were thus comparable within each location (Figs. 2 and 3) but were not directly comparable between the two locations. We therefore presented these data separately and no statistical comparisons were made between locations.

There were differences between the two locations in the average personal UVR dose received (Table 4, Fig. 3). Mothers in the rural location received an average of 4.53 MED (range 0.54 to 12.58 MED), which is almost six times the average daily UVR exposure of 0.78 MED (range 0.06 to 2.43 MED) received by mothers in the urban location.

**Table 4:** Personal UVR dose (MED), time outdoors and use of sun protection, Chinese Families and Children Study, 2012

	Urban			Rural		
	Mothers	Adolescents		Mothers	Adolescents	
		Male	Female		Male	Female
Average daily personal UVR dose (MED)^a^ (range)	0.78 (0.06, 2.43)	0.48 (0.06, 1.15)	0.81 (0.002, 2.87)	4.53 (0.54, 12.58)	2.16 (0.16, 8.17)	1.05 (0.03, 4.69)
Personal fraction %^b^	3.80%	2.34%	3.95%	22.38%	10.67%	5.19%
Average self-reported duration per day outdoors (minutes)^c^ (range)	81.1 (7.5, 315.2)	98.3 (7.5, 682.5)	72.9 (2.5, 158.7)	334.4 (30.0, 639.8)	147.9 (52.0, 612.5)	94.6 (30.0, 270.7)
Use of sun protection^d^:						
Sunscreen	10	0	0	7	1	5
Hats^e^	12	0	2	11	0	0
Gloves	10	0	0	6	1	0
Umbrella	5	0	0	0	0	0

^a^Minimal Erythemal Dose (MED)

^b^Estimated as the ratio between the average values of daily personal UVR dose divided by the average values of ambient UVR

^c^Average value of the group that completed 3 days of the sun diary

^d^Includes the use of any of the listed items during the 3-day study period

^e^Head partially or completely covered

Male and female adolescents in the rural location received about one half, and one quarter, respectively, of the average exposure of their mother. Male rural adolescents received approximately 4.5 times the MED of their urban counterparts, while rural female adolescents had only a slightly higher average MED than urban female adolescents. Male urban adolescents had the lowest average MED of any of the study participants.

Average self-reported time per day outdoors also differed significantly between the two locations, consistent with the average personal UVR dose. Time outdoors was highest in rural mothers, and at an average of 334 min, equated to over 5.5 h per day outdoors. By contrast, urban mothers spent on average less than 1.5 h outdoors. Of the adolescents, rural males reported the highest daily time outdoors (at 2.5 h) and urban females the least, at just over an hour. Urban males reported more time outdoors than urban females, however their personal UVR exposure recorded by dosimetry was less than that of urban females. There was a strong correlation between UVR exposure recorded by dosimetry and self-reported time outdoors (*r* = 0.59, *p* < 0.001). This correlation was observed primarily among mothers (*r* = 0.64, *p* < 0.001) and not among children (*r* = 0.08, *p* = 0.492). The correlation was also stronger in the rural north-eastern location (*r* = 0.54, p < 0.001), and not in the urban south-eastern location (r = 0.13, *p* = 0.217).

Some participants (primarily mothers) in both locations used some method of sun protection. Approximately one quarter of mothers in both locations reported wearing a hat at some time during the day, with a slightly smaller proportion using sunscreen and/or gloves. Umbrellas were used by a small number of urban mothers but were not used by rural mothers. In adolescents the most frequent method of sun protection was sunscreen, which was used by rural female adolescents. Hats were used by two urban female adolescents, while one rural male reported wearing a hat and/or sunscreen.

## Discussion

The current study is one of a small number of studies that report directly-measured UVR exposure in China, and one of the first to measure these exposures in mothers and their adolescent children using both polysulfone dosimetry and sun exposure diaries. The study has identified a strong regional variation in time spent outdoors and personal UVR exposure, particularly in mothers, and a variation in time outdoors and UVR exposure in adolescents, dependent on geographic region and sex.

Personal UVR exposure is influenced by environmental/geographical factors such as season, latitude, pollution, humidity and cloud cover, and by behavioral factors such as time spent outdoors (in turn, related to occupational and recreational activities), and use of sun-protection. During April to June, when our data was collected, mean daily temperatures in both locations were similar, with the urban (south-eastern) location being warmer and more humid. Interestingly, despite the difference in latitude, the average ambient UVR over the period of data collection was nearly identical in both locations (Table 3). While the values were not strictly comparable due to differences in the dates of measurement (taken at the beginning and end of data collection in each location), they are indicative of the two locations having a similar ambient UVR environment during April to June of 2012 (Fig. 2).

Many international studies linking UVR exposure with health outcomes use location or latitude of residence as a convenient proxy for personal UVR exposure [24–29]. Two studies in China used satellite measurements of cloud-adjusted ambient UVB in studies of cancer occurrence, mortality, and survival [30, 31]. Although based on small numbers, our findings, if replicated in other settings, suggest that latitude of residence may not be a good proxy for personal UVR exposure in all circumstances. Contrary to what might be expected, higher personal UVR exposures were received at the rural, higher latitude location, further from the equator. These findings align with those of a large multi-location Australian study, which showed that season and latitude modified the relationship between ambient UVR and personal UVR exposure [5]. Our findings suggest caution in using latitude and ambient UVR as indicators of UVR exposure, and attest to the importance of using personal, objective measures of exposure for prospective measurement when possible. This approach would not be feasible for retrospective exposure assessment.

Rural mothers received on average over 4.5 MED of UVR exposure per day, which is roughly equivalent to 22.4% of the total available UVR (due to the date inconsistency between measures of personal and ambient UVR exposure, this is based on estimates only) (Table 4). This is a substantial exposure that far exceeds the recommended occupational daily exposure limit of 1 MED per day for outdoor workers [32]. Indeed, several individual exposures were over 12 MED (Fig. 3), an exposure that equates to receiving over 60% of the total available UVR exposure for that day. As some rural mothers worked outdoors all day, this very high value could be expected. This high level of UVR exposure may have significant health implications, if sustained over the long term on unprotected skin or eyes [33]. We found only a relatively small percentage of rural mothers used sunscreen (17%) or hats (27%), hence many rural mothers may receive significant UVR exposure to the face. If further diary analysis reveals a lack of clothing cover (i.e. high skin exposure) on days of high UVR exposure, many rural mothers may be receiving a concerning personal UVR dose. Rural male adolescents, the group with the next-highest exposure, received an average of 10.7% of the available UVR, while rural female adolescents and all urban participants received a much smaller personal UVR dose and fraction.

The high UVR exposure experienced by rural mothers largely reflects their significant amount of time spent outdoors. Approximately 22% of rural mothers in our sample worked outside the home, primarily in farming or related occupations. By contrast, 80% of urban mothers worked outside the home, primarily in indoor occupations. While working does not necessarily equate with time spent outdoors, in smaller townships with rural economies, as in our north-eastern location, this is often the case. The rural lifestyle also presents more opportunities for outdoor exposure for mothers not in paid work, but tending to home farms. Our findings support those of a previous study that found that outdoor workers are exposed to approximately six to eight times more UVR than indoor workers and other groups [34]. Urban mothers, most of whom were in paid employment, did not spend much time outdoors during their activities of daily living. This may relate to the slightly higher temperature and increased humidity in the urban location compared with the rural location, and the fact that the main employment in the urban area was working in factories making cell phones components. Other studies have found that higher daily maximum temperatures can affect clothing and sun exposure behavior [5, 35–37]. In our study, occupation, weather factors, less opportunity for sun exposure, or deliberate sun avoidance for health, cultural or beauty reasons, may have played a part in the comparatively low UVR exposure of urban mothers.

Adolescents in both sites spent less time outdoors and had a lower personal UVR exposure dose than their mothers. At age 15–16 years, most Chinese adolescents are heavily involved in school, and the opportunity for recreational UVR exposure time is limited. Urban adolescent boys had the longest self-reported time outdoors but the minimum objectively-measured personal UVR exposure, among the urban population. This may be because they 1) over-reported their time spent outdoors due to misunderstanding the sun diary protocol, 2) covered the dosimeter with clothing while outdoors, or 3) removed outer clothing layers and the dosimeter while participating in outdoor activities, and did not replace the dosimeter on their arm as required. Rural male adolescents did, however, have a high personal UVR dose and spent significant time outdoors. This may have resulted from a requirement for outside-school assistance with farm or rural tasks or other outdoor activities. As the correlation between reported time outdoors and objectively-measured personal UVR exposure was generally poor in adolescents, further pilot testing is warranted to understand any difficulties experienced by adolescents in using the dosimeters and/or following the diary protocol. The wide variation in UVR dose and time outdoors within cohorts may result from a difference between weekend and weekday exposure (potentially more important for urban mothers and adolescents, where work or school activities may limit weekday exposure but allow weekend exposure), or a heterogeneity in occupational status (most relevant to rural mothers, where a small proportion were in paid indoor work while many of the remainder tend to farms or greenhouses on weekdays).

While our study numbers were small, our findings in mothers align with those of Yan et al., who showed that approximately 25% of Chinese participants wore hats and 21% wore sunscreen while outdoors, and that females were more likely to employ sun-protective behaviors than were males [15]. Studies of rural workers in other countries have also found hats to be used more frequently than sunscreen [38, 39].

A very small number of previous studies have used dosimetry to measured UVR exposure in Chinese participants. Liu et al. [18] used personal electronic dosimeters to study UVR exposure over four seasons in 62 Chinese school children and medical students in Shenyang (latitude 41 degrees), China, with simultaneous monitoring and recording of direct-ambient UVR dose and record-keeping of time outdoors. They observed that UVR doses of school children throughout the year were significantly higher than those of medical students. The average fraction of total available UVR received by participants varied depending on the season – in spring (the season of our study), school students received 3.77%, and medical students in Shenyang received 0.67% of the total available UV. The mean yearly MED value for school students was 0.278 (0.255 ~ 0.301) and for medical students was 0.095 (0.082 ~ 0.108). In our study, urban adolescent students received between 2.34% (males) and 3.95% (females) of the estimated total available UVR, which compares favourably with the findings of Liu et al. [18]. Rural adolescent students received a much higher proportion of the estimated total available UVR (10.67 and 5.19% for males and females, respectively), possibly because of a requirement for outside-school assistance with farm or rural tasks. Our findings, and those of others [18] show the importance of age and associated work and/or school responsibilities in determining personal UVR exposure in China.

An additional purpose of this study was to determine the feasibility of, and the compliance with data collection using dosimetry, and to consider what modifications may be required before wider rollout of the study. On this basis, the UVR measurement component of the study was successful, with high compliance and acceptability of the dosimetry and the diary, positive feedback, and minimal loss of data. The pilot study data support use of polysulfone dosimetry in larger studies as the method is cheap, reliable and has good compliance. A challenge noted for the future is to develop a dosimeter band that can easily accommodate changes in clothing and can fit over jackets. In addition, the band should eliminate the requirement for dosimeter attachment by Velcro dots, as some rural working mothers lost their dosimeters while performing heavy outdoor duties.

It is important to note that any measure of personal UVR exposure *reaching the skin* must include information on clothing and sun protection worn while outdoors, as this determines the body surface area exposed to UVR. UVR exposure of the skin is an important determinant of vitamin D production and skin cancer risk. An individual who spends much time outdoors may record a high personal UVR exposure dose by dosimetry, however if their body is fully covered with clothing or is well-protected by broad-spectrum sunscreen, the UVR cannot reach the skin to trigger vitamin D synthesis. In our study, detailed information on clothing worn while outdoors, use of sunscreen and other sun protection, and the timing of outdoor exposure each day was recorded in each individual’s sun diary, and will be the subject of future analyses. As clothing cover is impacted by ambient temperature, it is likely that the cooler average temperatures in both locations during the April and May data collections resulted in greater clothing cover (and less skin exposure to UVR) during these months. As temperature rises in June, skin exposure to UVR would be expected to increase, however time outdoors may decrease as people seek shade from the hot sun. As shown by others [5], an individual’s objectively-measured maximum personal UVR exposure dose (incorporating their skin exposure) may not always be achieved in summer, as other factors such as temperature - often dependent on latitude and altitude, can change personal behaviour.

Feedback from participants during data collection suggest the importance of developing a more culturally appropriate sun diary for use in Chinese populations, as the western images and clothing created confusion for some participants. Future versions of the diary should be updated to include facial masks, which are commonly used in the Chinese population against wind, dust, and air pollution. Consideration should also be given to including one or more questions about gradients in skin color, as there are large variations in skin color in the Chinese population and these may be relevant to sun exposure activities and patterns. In addition, future dosimetry studies in this cohort would benefit by assessing exposure for seven days each season, to allow investigation of the drivers of seasonal UVR exposure in different locations.

## Conclusions

This study contributes to the currently small body of knowledge about polysulfone dosimetry and personal UVR exposure in China. This study was limited by the small sample size, the short period and single season of UVR measurement (making individual measurements susceptible to unexpected adverse weather events) and the slightly different dates at which personal and ambient UVR were measured in the two locations. Although based on small numbers, our findings show the importance of geographic location, age, work/school responsibilities, and sex of the adolescents in determining personal UVR exposure in China. Our findings also confirm the usefulness of personal UVR dosimetry combined with a daily sun/clothing diary for measuring UVR exposure in Chinese populations, but suggest more work is necessary to 1) more appropriately tailor the diary to the Chinese population, and 2) improve the usability of dosimeters and diaries, especially for adolescents. Further pilot testing of dosimeter and diary protocols will be necessary before using these instruments on a large scale.

## Abbreviations

CDC: Centers for Disease Control and Prevention
CFCS: Chinese Children and Families Cohort Study
CIP: Community Intervention Program
IRB: Institutional Review Board
J/M^2^: Joules per meter squared
MCH: Maternal and Child Health
MED: Minimal Erythemal Dose
NCI: National Cancer Institute
nm: nanometer
QUT: Queensland University of Technology
US: United States
UVR: Ultraviolet Radiation
